# Assessment of the Auditory Handicap in adults with unilateral hearing loss

**DOI:** 10.1590/S1808-86942010000300018

**Published:** 2015-10-20

**Authors:** Patrícia Graciano Vicci de Araújo, Maria Fernanda Capoani Garcia Mondelli, José Roberto Pereira Lauris, Antonio Richiéri-Costa, Mariza Ribeiro Feniman

**Affiliations:** 1 Master's degree in The Science of Communication Disorders, the Sao Paulo University Hospital for the Rehabilitation of Craniofacial Anomalies (HRAC-USP). Speech therapist, Auditory Health Division, HRAC-USP; 2 Doctorate in The Science of Communication Disorders, HRAC-USP. Profa. Dra. of the Speech Therapy Department, Bauru Dentistry School, Sao Paulo University (FOB-USP); 3 Associate professor in the Pediatric Dentistry, Orthodontics and Collective Health Department, FOB-USP; 4 Associate professor. Geneticist at the HRAC-USP; 5 Associate professor in the Speech Therapy Department, FOB-USP. Auditory Health Division, HRAC-USP

**Keywords:** questionnaires, hearing loss, unilateral

## Abstract

Hearing impairment (HI) is characterized by unilateral hearing loss in one ear and can result in learning difficulties, language impairment and socio-emotional difficulties. To assess the perception of hearing handicap in adult subjects, patients with unilateral sensorineural HI, non-users of individual hearing aids.

**Materials and Methods:** Prospective study with 52 adult subjects with a mean of 34.5 years of age, from both genders (26 females, 26 males) with hearing loss: sensorineural unilateral, in varying degrees, responded to a questionnaire for assessing hearing handicap, and for that we used the Hearing Handicap Inventory for Adults (HHIA).

**Results:** We scored the subscales of the emotional and social/situational aspects, and we found 73.1% of the handicap present being mild, moderate and significant, but at a higher percentage (88.5%) in females.

**Conclusions:** The use of the questionnaire proved to be an effective procedure, because the unilateral HI may, not infrequently, compromise social and emotional aspects of the adult subject and the same degree of HI who can react differently, indicating that the wide variability in the perception of the hearing handicap is associated with non-audiological aspects.

## INTRODUCTION

Unilateral hearing loss is that which occurs in one ear; it affects mostly in males.^1^ According to Mariotto et al.,^2^ the main causes are mumps, ototoxicity, meningitis, noise-induced hearing loss, chicken-pox, cranial trauma, and unilateral sensorineural hearing loss of unknown cause. Laury et al.^3^ carried out a retrospective study of a database to study the etiology of unilateral hearing loss in children with otoacoustic emissions in the affected ear. They reported that the most common cause was cochlear nerve aplasia (73%), based on phenotype analysis of 11 subjects with unilateral hearing loss.

Unilateral hearing loss individuals find academic life harder, present language difficulties and face social and emotional hurdles.^4^ Thus healthcare professionals should monitor the hearing and the development of communication in these individuals, and should provide guidance to improve their chances of success while living with this type of hearing loss.^5^

The effects of unilateral hearing loss are as important as those caused by bilateral hearing loss in the presence of ambient noise. These individuals find it harder to understand speech, compared to normal hearing persons, even when the normal ear is well positioned towards the source of speech; spatial location of sources of sound is affected.^6^

Problems due to sensory deprivation may be minimized by using a personal sound amplification device (hearing aid); it makes it possible to retrieve ambient and speech sounds, which improves the ability to communicate.^7^

The decision to use a hearing aid begins with self-perception of the auditory handicap. According to a model proposed by the World Health Organization (WHO),^8^,^9^the auditory handicap is the negative impact of hearing loss on an individual's well-being and quality of life. The disadvantages imposed by hearing loss, which curtail an individual's psychosocial functioning, are compounded by non-auditory consequences of poor hearing. These include social and emotional manifestations that result from hearing loss, which may affect the individual, his or her family, and society. Measures to deal with this issue involve the type of hearing loss or disability, lifestyle, and the social, cultural and physical environment of each patient.

It has been said that of all sensory disabilities, loss of communication with other people may be one of the most frustrating consequences for individuals with hearing loss.^10^

Researchers^11^ have developed and standardized a questionnaire to assess the psychosocial effects of hearing loss in the elderly. The Hearing Handicap Inventory for the Elderly (HHIE) questionnaire consists of 25 questions divided into two scales (social/situational and emotional); it has been modified to provide an evaluation of the auditory handicap of adults, resulting in the Hearing Handicap Inventory for Adults (HHIA).^12^

The HHIA was standardized in a sample of 67 adults with hearing loss,^12^ and the test retest process demonstrated its reliability and internal consistency.^13^

In the present study, we chose to use the term handicap, rather than “disadvantage,” since it is a widely used English term in the scientific community and expresses better the concept to which it refers.

The purpose of this study was to assess self-perception of auditory handicap in adults with unilateral sensorineural hearing loss not using hearing aids.

## MATERIAL AND METHOD

The selection and assessment procedures of patients started after the institutional review board approved the study (Protocol 123/98) and patients signed the free informed consent form.

This cross-sectional contemporary cohort study included 52 adults of both genders (26 female, 26 male), with a mean age of 34.5 years.

The participant inclusion criteria were:

– Age: adult from 18 to 60 years;
– Hearing loss: unilateral sensorineural of varied degrees;
– Presence of hearing loss for more than one year;
– Non-users of hearing aids.

Of 5,000 regularly registered patients, 77 fit into these study criteria; 52 of these subjects agreed to participate in the study.

There were several etiologies according to the diagnoses made by the otorhinolaryngologist of the institution: cranial trauma (3), parotiditis (4), cholesteatoma (1), measles (1), sudden deafness (3), noise-induced hearing loss (1), vestibular syndrome (1), idiopathic (1), complication of chronic otitis media (1), otosclerosis (1), and of unknown cause (35).

The degree of hearing loss was given based on the audiometric thresholds at 500, 1000, 2000 and 4000 Hz: mild hearing loss (mean – 26 to 40 dBHL), moderate hearing loss (mean – 41 to 60 dBHL), severe hearing loss (mean – 61 to 80 dBHL), and profound hearing loss (mean over 81 dBHL), based on WHO criteria.14

The devices used for the exams were as follows: a model SD50 audiometer with HDA200 headphones, and a model SD30 impedance meter, both from Siemens.

The Portuguese version of the HHIA questionnaire15 (Annex 1) was used for evaluating the handicap; question 1S was adapted from: “Does difficulty in hearing make you use the telephone less than you wish?” to 1S: “Does your difficulty in hearing bring you problems when using the telephone?”

The HHIA is a self-assessment questionnaire of auditory handicap comprising 25 items, of which 13 deal with emotional aspects (E), and 12 deal with social and situational aspects (S). For each item or situation, subjects are asked to give one of the following answers: “yes” (4 points), “sometimes” (2 points) or “no” (0 points).

The questionnaire was applied individually by the investigator. Explanations were given if there were doubts about each question; care was taken not to induce answers, to avoid interviewer bias.

Scores ranged from zero to 100 percent; the score correlated with handicap perception, where a high score suggested a significant perception of hearing loss by the subject. Thus, a zero to 16 score indicated absent handicap; an 18 to 30 score indicated a mild handicap; a 32 to 42 score indicated a moderate handicap; and scores over 42 indicated a significant handicap.

Although this was not the purpose of the study, participants underwent a hearing aid selection and adaptation process, and again answered the HHIA questionnaire after six months of effectively using the hearing aid. This procedure was carried out to check whether the handicap could be changed by using a sound amplification device.

Spearman's correlation coefficient was used to verify whether the handicap correlated with age, degree and duration of hearing loss. The chi-square test was applied to verify any association between handicap and gender. The significance level in all tests was 5% (p<0.05).

**Annex 1 – english version:HEARING HANDICAP INVENTORY FOR ADULTS – HHIA - ALMEIDA K. (1998)15**

**Table:** 

	QUESTION	Yes	Sometimes	No
1S	“Does your difficulty in hearing bring you problems when using the telephone?”			
2E	Does a hearing problem cause you to feel embarrassed when meeting new people?			
3S	Does a hearing problem cause you to avoid groups of people?			
4E	Does a hearing problem make you irritable?			
5E	Does a hearing problem cause you to feel frustrated when talking to members of your family?			
6S	Does a hearing problem cause you difficulty when attending a party?			
7S	Does a hearing problem cause you to feel frustrated when talking to co-workers, clients, or customers?			
8E	Does a hearing problem cause you difficulty when going to the cinema or theater?			
9S	Do you feel handicapped by a hearing problem?			
10E	Does a hearing problem cause you difficulty when visiting friends, relatives, or neighbors?			
11S	Does a hearing problem cause you tdifficulty to listen/understand co-workers?			
12E	Does a hearing problem cause you to be nervous?			
13S	Does a hearing problem cause you to visit friends, relatives, or neighbors less often than you would like?			
14E	Does a hearing problem cause you to have arguments with family members?			
15S	Does a hearing problem cause you difficulty when listening to TV or radio Does a hearing problem cause you difficulty when listening to TV or radio?			
16S	Does a hearing problem cause you to go shopping less often than you would like?			
17E	Does any problem or difficulty with your hearing upset you at all?			
18E	Does a hearing problem cause you to want to be by yourself?			
19S	Does a hearing problem cause you to talk to family members less often than you would like?			
20E	Do you feel that any difficulty with your hearing limits or hampers your personal or social life?			
21S	Does a hearing problem cause you difficulty when in a restaurant with relatives or friends?			
22E	Does a hearing problem cause you to feel depressed?			
23S	Does a hearing problem cause you to listen to TV or radio less often than you would like?			
24E	Does a hearing problem cause you to feel uncomfortable when talking to friends?			
25E	Does a hearing problem cause you to feel left out when you are with a group of people?			

Total score:__________E Score:_________S score:________

## RESULTS

The emotional and social/situational subscales of 52 subjects who answered the HHIA questionnaire were scored. Table 1 shows the responses of the emotional subscale, presented as percentages, in which the highest score was given to the difficulty in hearing at the cinema or theater.

**Table 1:** Distribution of answers, as percentages, for each item of the emotional subscale

	YES	SOMETIMES	NO
Embarrassed	23,1%	19,2%	57,7%
Irritable	7,7%	55,8%	36,5%
Frustrated/family	7,7%	19,2%	73,1%
Cinema/theater	51,9%	15,4%	32,7%
Visit friends	13,5%	19,2%	67,3%
Nervous	25%	30,8%	44,2%
Discussions/family	5,8%	17,3%	76,9%
Upset	46,2%	36,5%	17,3%
Isolated	5,8%	13,5%	80,7%
Handicapped	21,2%	32,7%	46,1%
Depressed	9,6%	25%	65,4%
Uncomfortable/friends	26,9%	38,5%	34,6%
Left out	11,5%	26,9%	61,6%

Table 2 shows the responses of the social/situational subscale, presented as percentages, in which the highest score was given to the difficulty in using the telephone and going to parties; such situations induced the highest perception of handicap.

**Table 2:** Distribution of answers, as percentages, for each item of the social/situational subscale

	YES	SOMETIMES	NO
Telephone	40,4%	23,1%	36,5%
Avoid groups	9,6%	7,7%	82,7%
Parties	40,4%	34,6%	25%
Work	19,2%	44,2%	36,6%
Handicapped	7,7%	19,2%	73,1%
Work colleagues	21,1%	15,4%	36,5%
Visiting/less	1,9%	7,7%	90,4%
Radio/TV	21,2%	26,9%	51,9%
Buying/less	7,7%	5,8%	86,5%
Family dialogue/less	7,7%	5,8%	86,5%
Restaurant	23,1%	42,3%	34,6%
Radio/TV/less	11,5%	7,7%	80,8%

Table 3 shows the handicap classification, showing the distribution of subjects and almost homogeneous degree percentages. Table 4 shows the subjects and percentages relating gender and handicap. The presence of handicap was more prevalent in females.

**Table 3:** Distribution of subjects according to the classification of the auditory handicap

AUDITORY HANDICAP	N(%)
Absent	14(26,9%)
Mild	14(26,9%)
Moderate	11(21,2%)
Significant	13(25%)
TOTAL	52(100,0%)

**Table 4:** Distribution of subjects with a correlation between gender and the presence/absence of an auditory handicap

Gender Handicap	Female N(%)	Male N(%)
ABSENT	3(11,5%)	11(42,3%)
PRESENT	23(88,5%)	15(57,7%)
TOTAL	26(100,0%)	26(100,0%)

Chi-square Association Test = 4.78p=0.028

Table 5 illustrates the correlation between handicap and age, degree and duration of hearing loss.

**Table 5:** Correlation between the handicap and age, degree and duration of hearing loss

Variable	R	P
Age	0,27	0,049*
Degree of loss	0,16	0,255ns
Duration of loss	0,09	0,548ns

ns – statistically not significant correlation

* - statistically significant correlation (p<0.05)

Table 6 shows the distribution of subjects and percentages of the classification of auditory handicap after using a personal sound amplification device.

**Table 6:** Distribution of subjects and percentages relative to the classification of the degree of auditory handicap after using a personal sound amplification device

AUDITORY HANDICAP	N(%)
Absent	39(75%)
Mild	11(21,1%)
Moderate	2(3,9%)
Significant	0(0%)
TOTAL	52(100,0%)

## DISCUSSION

Assessing and analyzing the restrictions that individuals with unilateral hearing loss have in their daily lives provided us with knowledge about the effects of this condition on communicative, social and emotional aspects that make it possible for such persons to reflect about these factors and to understand their needs. Thus, considering that subjects with a similar audiometric profile may perceive their condition differently, and that traditional audiometric exams yield only basic information about a person's hearing ability, it becomes essential to assess communication difficulties and social and emotional consequences of hearing loss, which may be done by applying self-assessment questionnaires.^16^

Disadvantages due to hearing loss may appear in social and emotional situations. The distribution of the responses in the emotional subscale (Table 1) revealed that the perception of a handicap was associated with feeling upset because of hearing loss in 46.2% of subjects; this result concurs with that of other researchers^17^,^18^ where items related with frustration and isolation were the most common.

This shows the importance of applying these questionnaires, which make it possible to investigate the patient's perception about their communication difficulties; it also facilitates monitoring and helping these patients to identify their hearing needs, as well as those that are found in routine audiological assessments.^19^,^20^

The distribution of responses in each item of the social/situational subscale (Table 2) revealed that most of the “yes” and “sometimes” answers concerned difficulties of hearing in parties, at work and in restaurants. A common feature of these situations is the usual presence of ambient noise.

Subjects may face several speech understanding difficulties in noisy environments, as the number of cues decreases significantly, and they find themselves having to use only those cues available in that situation.^21^

The observed difficulty in gathering data about telephone use (Table 2) was unexpected, as normal hearing individuals also use a single ear to use the telephone. The laterality of subjects with unilateral hearing loss – answering the phone with his or her “preferred” side – might be an explanation, but this datum was not gathered. We found no similar data in the literature that could shed light on this discussion.

Table 3 shows the results of “absence of handicap” (scores from 0 to 16) and “presence of handicap” (scores over 16, including mild, moderate and significant cases), which revealed that 72.7% of the sample had some degree of auditory handicap, which we considered fundamentally important. These results concur with those of Newman et al. (1997)^17^ in which 74.6% of the sample had some degree of auditory handicap.

Correlating gender and the presence/absence of an auditory handicap (Table 4) revealed a lower handicap in males (42.3%) compared to females (11.5%); these findings were statistically significant. The present study, therefore, showed gender to be a source of variability in the self-perception of an auditory handicap, where females had a higher perception of their condition than males. According to the WHO8, age, gender, and psychosocial, cultural and environmental factors play important roles in the handicap.

We found no statistically significant correlations between the degree and the duration of hearing loss (Table 5); most subjects (96.2%) reported hearing loss lasting over a year. It should be said that this information is somewhat subjective, as unilateral hearing loss is often diagnosed accidentally and may go undiagnosed if there is no causal episode suggesting its presence. Thus, we may affirm that the duration is more closely related with the moment at which subjects perceived their hearing loss, rather than the moment it really started. Nevertheless, this information was investigated to verify for how long subjects lived with their hearing loss, with the aim of relating this aspect with possible manifestations of auditory handicap.

The HHIA questionnaire investigates whether hearing loss has altered the behavior of subjects in daily situations, as well as their attitude and response to this loss. Each subject reacts differently to hearing loss. Therefore, a mere pure tone analysis does not establish the extent of auditory handicap or the impact that hearing loss has on the daily lives of people.^22^,^23^,^24^

The HHIA has proven itself an excellent tool for predicting and confirming the performance of hearing impaired subjects in relation to communication difficulties in a variety of contexts; it may thus be of help in defining the amplification and rehabilitation needs, which are both directly linked with the patient's expectations and perception.

The importance of the HHIA questionnaire in this study was underlined by reapplying the questionnaire six months later in the same sample, after effectively using hearing aids, and finding significant changes in the scores; this finding suggests that subjects perceived better their hearing impairment.

In this study we hope to have provided additional information about the consequences of unilateral hearing loss in adults, and to foster an interest in further studies about this topic, with an emphasis on rehabilitation.

## CONCLUSION

Unilateral hearing loss may affect the social and emotional aspects of adults with this condition; these individuals require appropriate interventions.

Females had a higher perception of their handicap compared to males.

Subjects with the same degree of hearing loss may react differently, suggesting that the wide range of perception of an auditory handicap is associated with non-hearing factors.
